# Supplementary material for: Harnessing the IL-21-BATF Pathway in the CD8+ T Cell Anti-Tumor Response
Source: Cancers (Basel). 2021 Mar 12;13(6):1263. doi: 10.3390/cancers13061263 (PMC7998696; doi:10.3390/cancers13061263)
Supplement: Supplementary file 1 [file cancers-13-01263-s001.pdf]

cancers

MDPI

Supplementary Material: Harnessing the IL-21-BATF Pathway in the CD8+ T Cell Anti-Tumor Response

Paytsar Topchyan, Gang Xin, Yao Chen, Shikan Zheng, Robert Burns, Jian Shen, Moujtaba Y. Kasmani, Matthew Kudek, Na Yang and Weiguo Cui

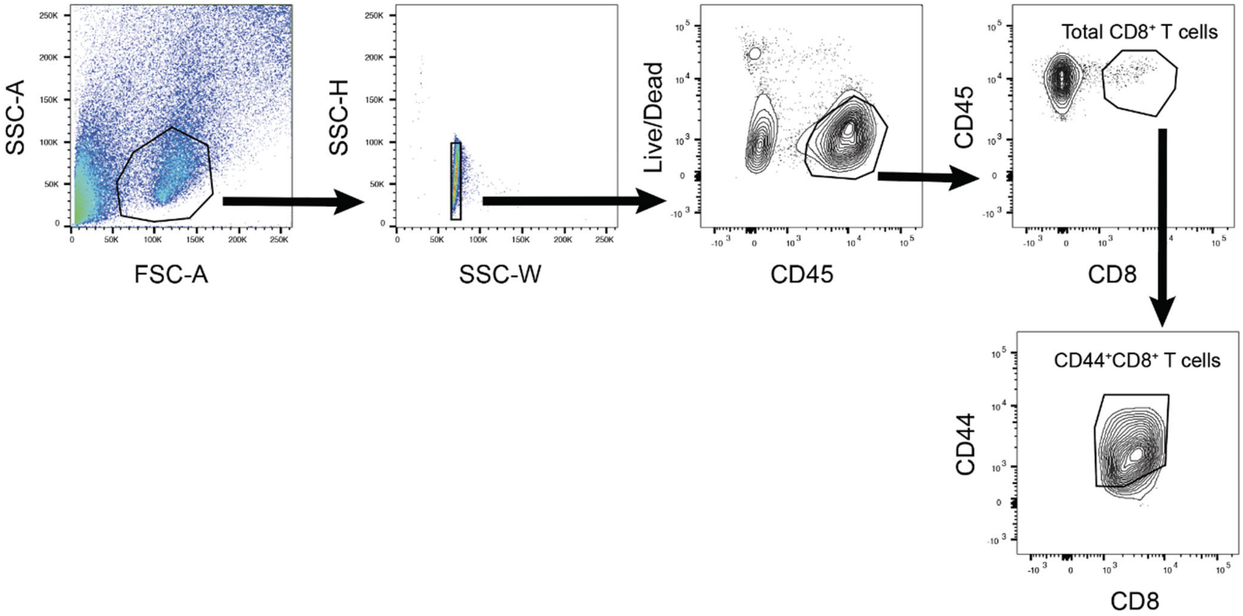

Figure S1. Gating strategy to assess tumor-infiltrating CD8+ T cells. Tumors of B16-GP33 tumor-bearing mice were harvested and immune cells were isolated. The flow cytometry panels above depict the gating strategy used to assess the CD8+ T cells in the tumor. First is the lymphocyte gate, followed by gating of single cells to exclude doublets, then gating on the live CD45+ leukocyte population. Then, the total CD8 T cell population is gated within the CD45 population, followed by looking at the activated CD44+ CD8+ T cell population.

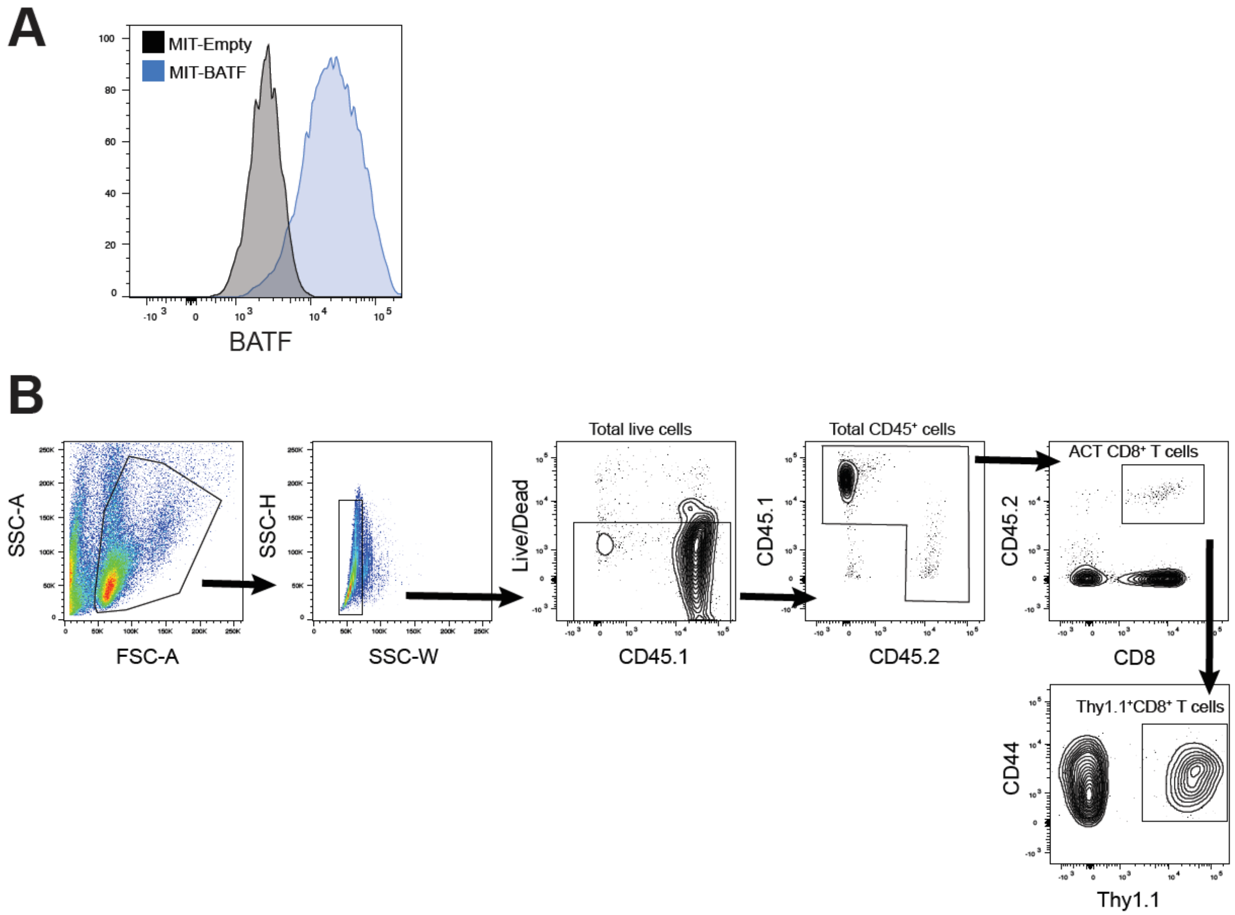

Figure S2. BATF retrovirally transduced CD8⁺ T cells. Pmel CD8⁺ T cells were activated and transduced with either MIT-Empty- or MIT-BATF-expressing retrovirus. (**A**) Forty-eight hours following transduction, flow cytometry was conducted to assess BATF expression levels in Thy1.1⁺ CD8 T cells. (**B**) Gating strategy to assess tumor-infiltrating CD8⁺ T cells in mice adoptively transferred with CD8 T cells retrovirally transduced with BATF. Tumors of B16-GP33 tumor-bearing mice (CD45.1 recipient mice) were harvested and immune cells were isolated. The flow cytometry panels above depict the gating strategy used to assess the CD8⁺ T cells in the tumor. First is the lymphocyte gate, followed by gating of single cells to exclude doublets, then gating on live cells. Total CD45⁺ cells were then selected and the adoptively transferred CD8 T cell population (CD45.2⁺) was selected. Thy1.1 expression of adoptively transferred cells was then assessed to identify retrovirally transduced CD8 T cells.

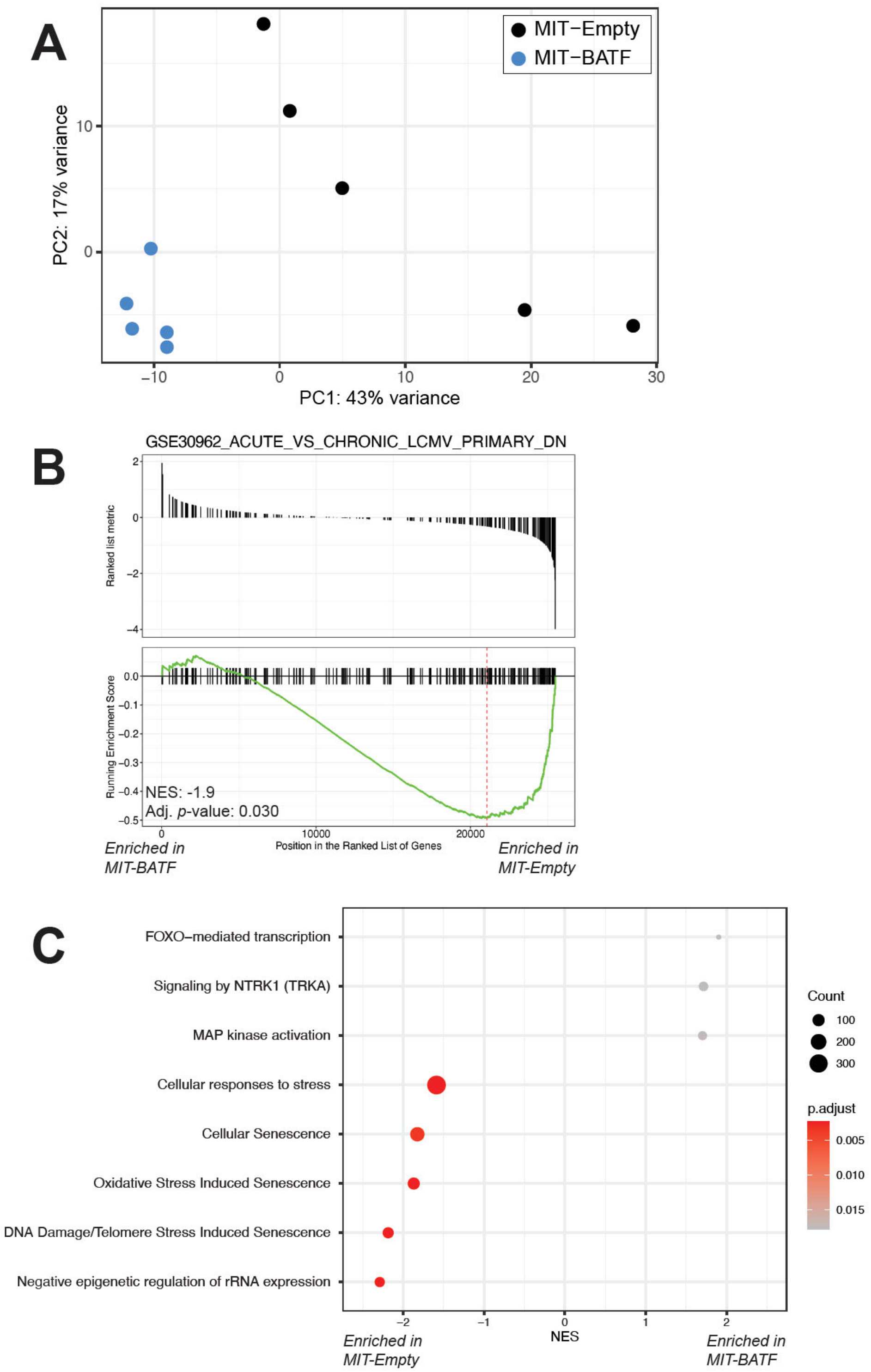

Figure S3. Tumor-infiltrating, adoptively transferred BATF-overexpressing CD8⁺ T cells are more resistant to cellular Scheme 16. F10 tumor-bearing mice were adoptively transFigure 8. T cells. One week following ACT, tumors were harvested and immune cells were isolated, stained, and FACS-sorted for Thy1.1⁺-expressing adoptively transferred CD8⁺ T cells. Bulk RNA sequencing was conducted on MIT-Empty and MIT-BATF cells. (**A**) Principle component analysis of samples. (**B**) Gene set enrichment analysis (GSEA) of genes downregulated in acute vs. chronic LCMV infection. (**C**) Reactome pathway analysis of relevant pathways. (*n* = 5 mice per group).
